# Chinese stroke patients with atrial fibrillation used Robert's age-adjusted warfarin loading protocol obtained good INR results within therapeutic range

**DOI:** 10.1038/s41598-023-45379-7

**Published:** 2023-10-25

**Authors:** Weiliang Luo, Xuanwen Luo, Suqin Chen, Jiming Li, Xiaodong Huang, Yu Rao, Wengsheng Xu

**Affiliations:** Department of Neurology, Huizhou Central People’s Hospital, No. 41, Eling North Road, Huizhou, 516001 Guangdong China

**Keywords:** Stroke, Drug safety

## Abstract

To assess whether Roberts’ age-adjusted warfarin loading protocol is effective in Chinese patients and whether the SAMeTT2R2 score can predict international normalized ratio (INR) control. Roberts’ protocol for warfarin titration was applied to patients with non-valvular atrial fibrillation (NVAF) complicated with ischemic stroke at the Department of Neurology between 2014 and 2019. Clinical and sociodemographic variables were recorded. A minimum of 1-year follow-up was used to calculate the time in therapeutic range (TTR) of the INR. A total of 94 acute ischemic stroke patients with NVAF were included in the study. Seventy-seven (81.9%) of the patients had attained stable INR (2.0–3.0) at the fifth dose, and 90.0% of the patients had achieved stable INR on the ninth day. Seventeen (18.1%) of the patients had an INR > 4 during dose-adjustment period. Patients with INR > 4 had significantly lower body weight (53.8 vs. 63.1 kg, *P* = 0.014), lower rate of achievement of stable INR (35.3% vs. 92.2%, *P* = 0.000), and lower rate of TTR ≥ 65% (23.5% vs. 70.1%, *P* = 0.001), but with no significant increase in bleeding risk. A total of 89 patients underwent long-term INR follow-up, of which 58 (65.2%) patients achieved TTR ≥ 65%. Patients with poor TTR had significantly lower body weight (56.3 vs. 63.7 kg, *P* = 0.020) and lower rate of stable INR achievement (64.5% vs. 89.7%, *P* = 0.002). All 94 patients had SAMeTT2R2 score ≥ 2. There was no linear association between SAMeTT2R2 score and the rate of TTR ≥ 65% (*P*_*trend*_ = 0.095). Chinese ischemic stroke patients with NVAF on warfarin can safely and quickly achieve therapeutic INR using Roberts’ age-adjusted protocol and can obtain a good TTR. Lower body weight may be a predictor of poor TTR and INR > 4. Patients who have not attained stable INR after adjusting the dose five times are at high risk for poor TTR. SAMeTT2R2 score may not predict TTR in Chinese ischemic stroke patients with NVAF.

## Introduction

Globally, atrial fibrillation (AF) is the most common cardiac rhythm disorder, and the prevalence rate of AF increases with age^1^. According to a large-scale epidemiological study conducted in 2008, the estimated prevalence of AF in China is approximately 0.77%, and among which, non-valvular atrial fibrillation (NVAF) accounts for 65.2% of the cases in the general population and 75.93% of the cases in the hospitalized population^2^. AF is associated with a five-fold higher risk of stroke^3,4^. Cerebral embolism caused by NVAF accounts for 13%–26% of all cases of ischemic stroke^5,6^. Anticoagulant therapy reduces the risk of AF-related stroke and all-cause mortality^7^. Therefore, the practice guidelines recommend oral anticoagulants, either vitamin K antagonists (VKAs) or non–vitamin K oral anticoagulants (NOACs), for stroke prevention in AF patients who are at a high risk of thromboembolism^8^.

Among patients on warfarin therapy, > 65% of time in therapeutic range (TTR) of the international normalized ratio (INR) is considered indicative of good treatment effectiveness as well as safety^9^. Though warfarin remains the most prescribed anticoagulant agent in China, oral anticoagulant (OAC) treatment rate in China is much lower than that in the developed countries^10^. This is largely attributable to the challenges associated with dose-adjustment and the risk of bleeding which constrains the willingness of patients to opt for these drugs^11^. Determining the optimal approach for initiation and dose-adjustment to rapidly and safely achieve the therapeutic INR is a key challenge in clinical settings. Fennerty’ method of initiating 10 mg warfarin can help to achieve the therapeutic INR effectively in relatively young patients (average age: 52 years), but causes excessive anticoagulation and bleeding in elderly patients^12,13^. Robert et al. proposed an age-adjusted warfarin loading protocol based on Fennerty’s method, which was found to be safe and effective in Australian patients^14^. Although our previous study has verified the short-term applicability of this approach in the Chinese population^15^, its long-term applicability requires further investigation.

The SAMeTT2R2 score (S: Sex [female] [1 point]; A: age < 60 years [1 point]; Me: Medical History [> 2 of the following comorbidities: hypertension, diabetes, coronary artery disease/myocardial infarction, peripheral arterial disease, congestive heart failure, previous stroke, pulmonary disease, hepatic or renal disease] [1 point]; T: Treatment [interacting drugs e.g., amiodarone for rhythm control] [1 point]; T: Tobacco use (within 2 years) [2 points]; and R: Race [non-white] [2 points]) was developed as a tool to predict poor INR control in patients on warfarin^16^. Patient's with SAMeTT2R2 score ≥ 2 are considered to be at risk of poor TTR on warfarin, and NOACs are recommended for these patients as an alternative^16^. Since Chinese represent a non-Caucasian population, the minimum score for Chinese patients with stroke will be 2 points. However, according to our clinical experience, Chinese stroke patients with SAMeTT2R2 score of ≥ 2 can still obtain good TTR with warfarin. In another trial conducted in Asian population, SAMeTT2R2 score was not found to predict INR control^17^. Therefore, in this study, we investigated whether SAMeTT2R2 score and Roberts’ age-adjusted protocol are applicable to the Chinese stroke population.

## Materials and methods

### Ethics

Ethics approval (LLBA201301) for this study was obtained from the Huizhou Central People’s Hospital Affiliated to Guangdong Medical University. Written informed consent was obtained from all patients prior to their enrollment. All methods were carried out in accordance with relevant guidelines and regulations. All research was performed in accordance with the Declaration of Helsinki.

### Inclusion criteria and treatment regime

This was a prospective observational cohort study. Adult patients with NVAF complicated with acute ischemic stroke who were admitted to the Department of Neurology at the Huizhou Central People’s hospital between January 1, 2014 and December 31, 2019 were eligible for inclusion. The diagnostic criteria for ischemic stroke was based on the guidelines of the Neurology Branch of the Chinese Medical Association (2010)^18^. The diagnosis of AF was consistent with the 2001 ACC/AHA/ESC guidelines^19^. Newly diagnosed patients with paroxysmal AF patients were excluded from this analysis, since these patients were reluctant to receive anticoagulation therapy.

Patients included in the study cohort were allowed to take warfarin (OrionMarevan, 3 mg/tablet, Orion Corporation, Finland). The indication for anticoagulation was CHA2DS2-VASc score ≥ 2 points (3 points for female) according to the guidelines^19^. Patients receiving warfarin followed the Roberts’ age-adjusted loading protocol for dose-adjustment (Table 1).

**Table 1:** Roberts’ age-adjusted warfarin initiation protocol.

Day	INR	Dose for age (mg)			
		≤ 50y	51–65 y	66–80 y	> 80 y
1	< 1.4	10	9	7.5	6
2 (16 h after 1st dose)	≤ 1.5	10	9	7.5	6
	≥ 1.6	0.5	0.5	0.5	0.5
3 (16 h after 2nd dose)	≤ 1.7	10	9	7.5	6
	1.8–2.3	5	4.5	4	3
	2.4–2.7	4	3.5	3	2
	2.8–3.1	3	2.5	2	1
	3.2–3.3	2	2	1.5	1
	3.4	1.5	1.5	1	1
	3.5	1	1	1	0.5
	3.6–4.0	0.5	0.5	0.5	0.5
	> 4.0	0	0	0	0
4 (16 h after 3rd dose)	≤ 1.5	10–15	9–14	7.5–11	6–9
	1.6	8	7	6	5
	1.7–1.8	7	6	5	4
	1.9	6	5	4.5	3.5
	2.0–2.6	5	4.5	4	3
	2.7–3.0	4	3.5	3	2.5
	3.1–3.5	3.5	3	2.5	2
	3.6–4.0	3	2.5	2	1.5
	4.1–4.5	omit next days’ dose, then			
		1–2	0.5–1.5	0.5–1.5	0.5–1
	> 4.5	withhold dose			

*INR* international normalized ratio.

Patients routinely received warfarin doses at 8:00 Hrs each day during titration protocol. Blood samples for INR were obtained between 07:00 to 08:00 h the next morning.

The timing of initiation of anticoagulation was dependent on the severity of the stroke following the 2013 guidelines of the European Heart Rhythm Association (EHRA)^20^, i.e., on the 3rd, 6th, and 12th day after the onset of mild stroke (National institutes of health stroke scale, NIHSS < 8), moderate stroke (8 ≤ NIHSS ≤ 15) and severe stroke (NIHSS > 15), respectively. Before anticoagulation, cranial computed tomography (CT) or magnetic resonance imaging (MRI) were performed to exclude intracranial hemorrhage^20^. In case of increase in the NIHSS score by ≥ 3 points or development of new neurological symptoms during anticoagulation, cranial CT or MRI was performed to confirm whether there was intracranial hemorrhage or progression of infarction. Anticoagulation therapy (warfarin) was terminated in the event of confirmation of intracranial hemorrhage, gastrointestinal bleeding, or hemoptysis.

Patients were asked not to take any traditional Chinese medicines while using warfarin^21^, since some traditional Chinese medicines such as Salvia miltiorrhiza, Ginkgo biloba, and Angelica can increase INR. There are too many kinds of traditional Chinese medicines for us to list them one by one, so we cannot provide detail criteria to define concomitant Chinese herbal medicine. The impact of most Chinese herbal medicines on warfarin is still unclear^22^.

### Exclusion criteria

The exclusion criteria were: patients who did not complete the protocol (daily doses for 4 days with subsequent INRs) or those who deviated from the doses stated in the protocol; patients who had received vitamin K in the preceding 2 weeks or warfarin in the preceding week; patients with history of advanced malignancy or those who developed diarrhea and/or vomiting during the study protocol; patients with hyperthyroidism and those receiving enteral or nasogastric feeding. Other exclusion criteria included significant valvular heart disease (i.e., prosthetic heart valve, rheumatic heart disease), or less than 10 retrievable INR after titration protocol and an additional 2 days of empiric adjustment, or warfarin interruption, or missing follow up data. Patients with other co-existing diseases (such as systemic lupus erythematosus or tuberculosis) that necessitated the use of drugs which significantly interfere with warfarin metabolism, were all excluded from the study.

The main outcomes were the number of doses required to achieve a stable INR (detected by STAR MAX automatic coagulation analyzer, Stago, France) and the percent of patients with TTR ≥ 65%. A stable INR was defined as either the first of the two consecutive INRs in the range of 2–3 or the first measurement in the therapeutic range when the previous or subsequent INR varied no more than 0.5 points outside the target range according to Roberts’ protocol^14^. Patients were required to recheck INR once a month after attaining stable INR. If three or more consecutive readings of stable INR were attained, the INR was rechecked once every 3 months. Patients got ≥ 4 times of follow-up INR for the calculation of TTR. The secondary endpoints were recurrent cerebral infarction or intracranial hemorrhage.

### Statistical analysis

Normally distributed continuous variables were expressed as mean ± standard deviation, and non-normally distributed continuous variables were expressed as median and percentile. Time to reach a stable therapeutic INR was examined using Kaplan–Meier method. Student’s *t* test was used to compare parametric data, while Mann–Whitney U test was used to compare non-parametric data. Chi squared test, Pearson Chi-squared, or Fisher's exact test was applied to compare the frequency data between the two groups based on the sample size and the number of cells with value < 5. P values < 0.05 were considered indicative of statistical significance. Data analyses were performed using IBM SPSS Statistics V22.0 (IBM Corp., Armonk, NY, USA).

## Results

**Figure 1:**
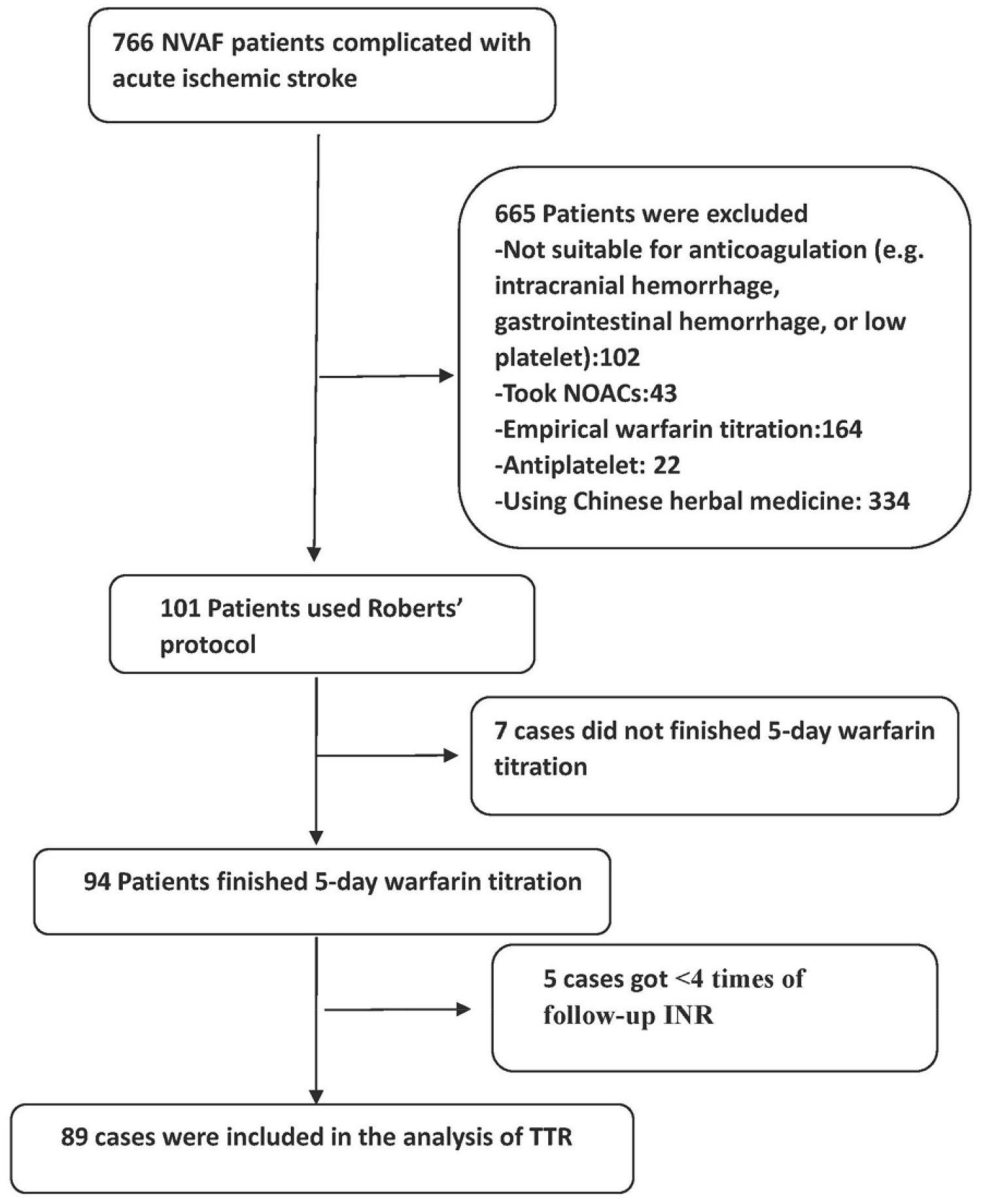
Flow chart illustrating the patient-selection process. There were 766 acute ischemic stroke patients with NVAF. Based on the inclusion and exclusion criteria, eventually 89 cases were included in the analysis of TTR.

The flow chart of the included cases is shown in Fig. 1. During our research period, there were 766 acute ischemic stroke patients with NVAF, and 255 (33.3%) of the cases used warfarin for anticoagulation, of which, 101 patients who met the inclusion criteria used Roberts’ protocol for warfarin titration. Among these patients, seven cases did not finished 5-day warfarin titration, 94 patients finished 5-day warfarin titration, and five cases got < 4 times of follow-up INR. Thus, 89 cases were included in the analysis of TTR.

The characteristics of the study population are summarized in Table 2. The average age of the patients was 67.9 ± 11.6 years; 48.9% patients were in the age-group of 66–80 years, and 28.7% were in the age-group of 51–65 years. The male/female ratio in our cohort was 53/41 1.3/1. The proportion of patients with chronic diseases, such as hypertension, diabetes, congestive heart failure, and renal failure (maintenance dialysis), was 38.3%, 13.8%, 4.3%, and 1.1%, respectively.

**Table 2:** Comparison between two groups with INR > 4 and ≤ 4 within 5 days of titration.

Parameter	All patients (n = 94)	Patients with INR ≤ 4 (n = 77)	Patients with INR > 4 (n = 17)	*P*
Age (years)	67.9 ± 11.6	68.2 ± 11.8	66.6 ± 11.6	0.942
≤ 50	7 (7.5%)	5 (6.5%)	2 (11.8%)	0.886
51–65	27 (28.7%)	24 (31.2%)	3 (17.7%)	
66–80	46 (48.9%)	36 (46.8%)	10 (58.8%)	
> 80	14 (14.9%)	12 (15.6%)	2 (11.8%)	
Weight (kg)	61.6 ± 11.0	63.1 ± 11.3	53.8 ± 11.0	0.014
Male/female (n)	53/41	46/31	7/10	0.367
Diabetes mellitus	13 (13.8%)	11 (14.3%)	2 (11.8%)	1.000
Albumin (g/L)	37.1 ± 3.0	37.3 ± 3.1	36.0 ± 3.0	0.121
Creatinine (µmol/L)	86.7 ± 26.9	83.7 ± 27.0	104.2 ± 26.9	0.233
Maintenance dialysis	1 (1.1%)	0 (0%)	1 (5.9%)	0.181
Hypertension	36 (38.3%)	27(35.1%)	9 (52.9%)	0.170
Congestive heart failure	4 (4.3%)	3 (3.9%)	1 (5.9%)	1.000
NSAIDs or alcohol	4 (4.3%)	4 (5.2%)	0 (0%)	1.000
Amiodarone	3 (3.2%)	3 (3.9%)	0 (0%)	1.000
SAMeTT2R2	3 (2–3)	3 (2–3)	3 (3–4)	0.328
HAS-BLED	2 (2–3)	2 (2–3)	2 (1.5–3.5)	0.654
CHA2DS2-VASc	4 (3–5)	4 (3–5)	4 (3–6)	0.599
Get stable INR at the 5-day	77 (81.9%)	71 (92.2%)	6 (35.3%)	0.000
Patients with follow-up INR more than 4 times	89 (94.7%)	72 (93.5%)	7 (41.9%)	0.000
Times of INR follow-up	7 (5–11)	7 (5–11)	5.5 (5–23.75)	0.673
TTR ≥ 65%	58 (65.2%)	54 (70.1%)	4 (23.5%)	0.001

*NSAIDs* nonsteroidal anti-inflammatory drugs, *INR* international normalized ratio, *TTR* time in therapeutic range.

**Figure 2:**
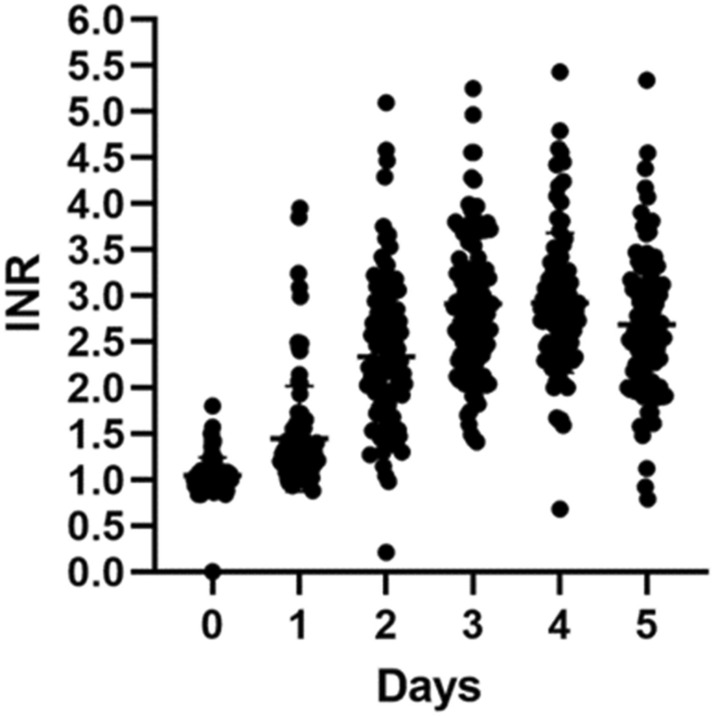
INR changes during titration. Day 0 represents the baseline INR value before warfarin use. INR value on day 1 was detected 24 h after the first dose of warfarin, and so on. The area in the figure represents the number of cases and shows the median and quartile.

**Figure 3:**
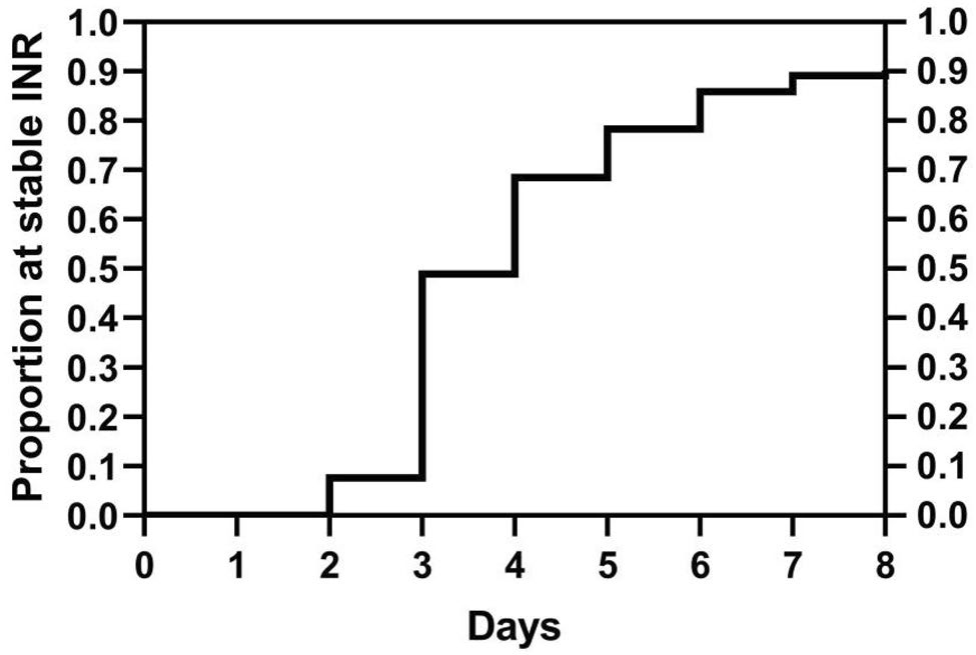
Proportion of patients with stable INR. Kaplan–Meier curve was applied to analyze the proportion of patients attaining stable INR at different days.

The dynamic changes in INR are shown in Fig. 2. Most patients achieved stable INR at the fourth and fifth dose. Seventy-seven (81.9%) patients achieved stable INR on the fifth day. On the ninth day, approximately 90% patients achieved stable INR (Fig. 3). Fifty-eight (65.2%) patients had a TTR ≥ 65%. There were 17 cases with INR > 4 at the fifth dose of warfarin. Compared with patients with INR ≤ 4, the patients with INR out of range had significantly lower body weight (53.8 vs. 63.1 kg, *P* = 0.014), lower rate of achievement of stable INR achievement (35.3% vs. 92.2%,* P* = 0.000), and lower rate of TTR ≥ 65% (23.5% vs. 70.1%, *P* = 0.001). There was no significant differences between these two groups with respect to other clinical variables, including age, albumin level, systemic diseases, use of amiodarone, or SAMeTT2R2 score (Table 2).

In order to identify the possible factors affecting TTR, comparison of patients with TTR ≥ 65% and < 65% was made (Table 3). Patients with TTR < 65% were found to have significantly lower weight (56.3 vs. 63.7 kg, *P* = 0.020), and lower rate of stable INR attainment at the fifth dose (64.5% vs. 89.7%, *P* = 0.002). However, there was no between-group difference with respect to rate of recurrent stroke or hemorrhage rate (*P* = 0.959). We also performed a linear by linear association analysis between the SAMeTT2R2 score and TTR to determine whether SAMeTT2R2 score can predict poor INR control. The results showed no linear association between SAMeTT2R2 score and the rate of TTR ≥ 65% (Table 4, *P*_*trend*_ = 0.095), indicating that SAMeTT2R2 score was not a predictor of TTR in our Chinese cohort. In patients who achieved stable INR, there was no correlation between the number of days the patients achieved stable INR and whether TTR was achieved. Although the day when patients reached stable INR was not associated with TTR, but patients who did not achieve stable INR showed significant association with poor TTR (Table 5, *P*_*trend*_ = 0.000).

**Table 3:** Comparison between patients with TTR ≥ 65% and < 65%

Parameter	TTR ≥ 65% (n = 58)	TTR < 65% (n = 31)	*P*
Age (years)	68.2 ± 11.4	68.0 ± 11.6	0.926
≤ 50	3	3	0.963
51–65	20	7	
66–80	26	18	
> 80	9	3	
Weight (kg)	63.7 ± 11.3	56.3 ± 9.3	0.020
Male/female (n)	34/24	15/16	0.355
Diabetes mellitus	8	4	1.000
Albumin (g/L)	37.4 ± 2.9	36.4 ± 3.6	0.295
Creatinine (µmol/L)	83.4 ± 21.8	92.1 ± 32.1	0.139
Hypertension	22	12	0.943
Congestive heart failure	2	2	0.909
NSAIDs or alcohol	2	1	1.000
SAMeTT2R2	3 (2–3)	3 (3–4)	0.151
HAS-BLED	2 (2–4)	2 (2–3)	0.335
CHA2DS2-VASc	4 (3–5)	4 (4–4)	0.782
Achieved stable INR at the 5-day	52 (89.7%)	20 (64.5%)	0.002
Times of INR follow-up	8.5 (6–11)	6 (5–11)	0.130
Recurrent stroke	4	3	0.959

*NSAIDs* nonsteroidal anti-inflammatory drugs, *INR* international normalized ratio, *TTR* time in therapeutic range.

**Table 4:** Correlation between TTR and SAMeTT2R2 score.

SAMeTT2R2	TTR ≥ 65%	TTR < 65%	*P*_*trend*_
2 (n = 24)	19 (79.2%)	5 (20.8%)	0.095
3 (n = 48)	29 (60.4%)	19(39.6%)	
4 (n = 11)	8 (72.7%)	3 (27.3%)	
5 (n = 4)	1 (25.0%)	3 (75.0%)	
6 (n = 2)	1 (50.0%)	1 (50.0%)	

*TTR* time in therapeutic range.

**Table 5:** Correlation between Time to achieve stable INR and TTR.

Time to achieve stable INR	TTR ≥ 65%	TTR < 65%	*P*_*trend*_
Not attaining stable INR (n = 8)	0	8	0.000^a^
D2 (n = 7)	7	0	0.079^b^
D3 (n = 38)	28	10	
D4 (n = 18)	13	5	
D5 (n = 9)	4	5	
≥ D6 (n = 9)	6	3	

*INR* international normalized ratio, *TTR* time in therapeutic range.

^a^Comparison between the sub groups attaining stable INR and not attaining stable INR.

^b^Comparison within those achieving stable INR at different days.

## Discussion

Although the efficacy and safety of NOACs is better than that of warfarin^23^, the higher cost of NOACs^24^, the lack of monitoring indices, and the lack of reversal agents limited their application^24–26^. Warfarin is still the most widely prescribed anticoagulant in developing countries, including China^10,27^. Development of a warfarin titration protocol that is suitable for the Chinese population is a key imperative. Our previous pilot study has verified the short-term applicability of Roberts’ method in Chinese patients^15^. In this larger study with a longer duration of follow-up, we found that the overall proportion of patients with stable therapeutic INR was approximately the same as in the previous report^14^, but the proportion of patients with INR > 4 was much higher than that in Roberts's report^14^. Moreover, long-term follow-up INR showed that 65.2% of the patients had good TTR (≥ 65%), which was a little bit lower than previous reports^28,29^.

SAMeTT2R2 score has been shown to predict patients with low or high probability of poor INR control prior to initiation of warfarin treatment based on clinical risk factors^16^. Patients with AF who have SAMeTT2R2 score of 0–1 are considered to do well on VKAs. Conversely, SAMeTT2R2 score ≥ 2 predicts poor INR control on warfarin and NOACs are recommended for these patients to achieve acceptable anticoagulation therapy control^16^. Though SAMeTT2R2 score has been shown to be a good predictor in the Caucasian population^16,30^, its predictive value is very limited in Asian populations due to the fact that non-Caucasian status is immediately assigned 2 points. It is not surprising to find non-Caucasian patients with SAMeTT2R2 score ≥ 2 but with average TTR higher than 78%^31^ or those with SAMeTT2R2 score ≥ 3 but with TTR higher than 70%^32^. Our study found that Chinese stroke patients with AF who obtained 2 or more points in SAMe-TT2R2 score could still achieve good TTR when using warfarin. Our results support the suggestion that SAMeTT2R2 score may not be predictive of TTR, especially in Chinese patients^33^.

The initial dose of warfarin plays an important role for the time taken to reach a stable INR and even determining the stable maintenance dosage. However, prescribing warfarin individually has always been a great challenge for physicians. The pharmacokinetics of warfarin show considerable inter-individual variability, and is affected by a multitude of factors including the balance of the four vitamin K-dependent clotting factors, vitamin K status, age, and warfarin dose; therefore, development of an optimal approach to initiate and calibrate the drug dosage to rapidly and safely achieve a stable target INR is critical.

There are several published protocols for initiation of therapeutic or prophylactic warfarin therapy, either aiming at rapid or slow attainment of a therapeutic INR^34,35^. Initiation with 10 mg or 5 mg dose of warfarin have been most studied in the past two decades. Comparison studies of 5-mg and 10-mg loading dose showed that 5-mg dose of warfarin produces less excess anticoagulation but takes longer time to reach therapeutic INR^35^. Since age is a independent factor that independently affects the dose effect of warfarin, Roberts et al. proposed the age-adjusted warfarin loading protocol based on the Fennerty method^14^. In this protocol, the warfarin dosage on a particular day is adjusted according to the INR on the preceding day. Compared to 10 mg on day-one and 5 mg on day-two regimen, the mean time to an in-range INR was significantly longer (0.8–1 day longer) for patients on the age-adjusted regimen; however, the age-adjusted regimens were associated with significantly fewer patients with out-of-range INR^36^. The age-adjusted loading protocol offers important clinical advantages over the Fennerty regimen with respect to individualization of dose and safety in the induction stage. Additionally, it should be noted that based on our current statistical data, only about 33.3% of Chinese acute ischemic stroke patients with NVAF took warfarin for anticoagulation. Although the rate on warfarin was higher than previous reports^37^, it was still far lower than developed countries. This is highly relevant to China's special national conditions. Chinese people prefer to use traditional Chinese medicine, with a usage rate of over 50% among stroke patients. Due to the complexity and uncertainty of traditional Chinese medicine ingredients, they may affect the metabolism of warfarin. Studies have shown that traditional Chinese medicine, such as Salvia miltiorrhiza or Ginkgo biloba, could interfere the stability of INR^38^. During the enrolling stage, many patients refused to give up using traditional Chinese medicine or could not promise not to take traditional Chinese medicine in the future,Therefore, this study excluded patients who used traditional Chinese medicine, and resulted in a low enrollment rate.

Our study indicated that Roberts’ protocol was generally safe and effective for Chinese patients. The much higher proportion of INR > 4 was mainly due to the lower body weight of Chinese population compared with Caucasians. Further, INR not reaching a stable state on day 5 and lower body weight were associated with poor TTR. We suggest that a more detailed and individualized dose adjustment based on weight may be required for application of Roberts’ protocol to the Chinese population in order to achieve therapeutic INR quickly and safely. Alternatively, patients who do not achieve stable INR over 5 doses may not do well on warfarin, and replacement of warfarin with NOACs may be reasonable for these patients.

### Limitations

Some limitations of our study should be considered while interpreting the results. This was a single-center study with a small sample size because a considerable number of patients did not receive anticoagulation treatment but using traditional Chinese medicine as alternate were excluded in this study. There was no comparison with other initiation loading protocol at the same time. Since many drugs are known to affect the metabolism of warfarin, this study did not analyze the usage of all other drugs in each patient; therefore, there may be uncontrolled confounders in our study. More robust multicenter anticoagulation studies are required to identify better warfarin initiation protocol and predictors of poor INR control.

## Conclusions

Roberts’ age-adjusted warfarin loading protocol may be applicable to most of the Chinese population with a high rate of achievement of stable INR achievement and good TTR. In this study, low body weight was associated with INR > 4 and poor TTR. SAMeTT2R2 score was not predictive of TTR in Chinese patients.

## Data Availability

All data requests should be submitted to the corresponding author for consideration. Access to anonymized data may be granted following review.
